# Ten years of major equestrian injury: are we addressing functional outcomes?

**DOI:** 10.1186/1752-2897-3-2

**Published:** 2009-02-19

**Authors:** Jill E Ball, Chad G Ball, Robert H Mulloy, Indraneel Datta, Andrew W Kirkpatrick

**Affiliations:** 1 The Trauma Program, University of Calgary, Calgary, AB, Canada; 2 Department of Surgery, University of Calgary, Calgary, AB, Canada

## Abstract

**Background:**

Horseback riding is considered more dangerous than motorcycle riding, skiing, automobile racing, football and rugby. The integral role of rehabilitation therapy in the recovery of patients who have sustained a major horse-related injury is previously not described. The goals of this paper were to (1) define the incidence and pattern of severe equestrian trauma, (2) identify the current level of in-patient rehabilitation services, (3) describe functional outcomes for patients, and (4) discuss methods for increasing rehabilitation therapy in this unique population.

**Methods and results:**

A retrospective review of the trauma registry at a level 1 center (1995–2005) was completed in conjunction with a patient survey outlining formal in-hospital therapy. Forty-nine percent of patients underwent in-patient rehabilitation therapy. Injuries predictive of receiving therapy included musculoskeletal and spinal cord trauma. Previous injury while horseback riding was predictive of not receiving therapy. The majority (55%) of respondents had chronic physical difficulties following their accident.

**Conclusion:**

Rehabilitation therapy is significantly underutilized following severe equestrian trauma. Increased therapy services should target patients with brain, neck and skull injuries. Improvements in the initial provision, and follow-up of rehabilitation therapy could enhance functional outcomes in the treatment resistant Western equestrian population.

## Background

Horseback riding, both recreational and work related, are popular activities in Alberta. Over 470,000 people are employed exclusively within the Canadian equestrian industry [1]. In addition, Alberta hosts the largest rodeo in the world and possesses over 50% of the 854,032 horses in the country [1].

Unfortunately, horse-related activities are also a significant contributor to major injury. When compared to motorcycle riding, equestrian activity has a higher hospital admission rate of 0.14/1000 hours versus 0.49/1000 hours [2,3]. Consequently, horseback riding is considered more dangerous than motorcycle riding, skiing, automobile racing, football and rugby [3-6]. The riding position itself creates this high-risk situation. Horseback riding elevates the rider's head 3 meters above the ground on an animal that can easily weigh 500 kg or more, kick with a force of nearly 1 ton, and run at speeds of 65–75 km/h. In addition, horses are less predictable than either a motorcycle or a racecar.

Patients sustaining major injuries from horseback riding often require rehabilitation therapy. The therapy team may include a physiatrist, occupational therapist, physical therapist, speech therapist, as well as various assistants. In the acute care setting, the therapy team collaborates to assist patients in regaining their functional independence through client-centred and evidence-based practice methods [7]. Client-centered practice requires them to understand all activities that bring meaning and purpose to their patient's lives [8]. For the equestrian population, this typically translates into getting "back in the saddle again".

Although previous studies have outlined the long-term consequences of equestrian injuries [9,10], this is the first project specifically designed to investigate the provision, utility and outcomes of rehabilitation therapy during a hospital admission. Furthermore, this study describes patients with a mean injury severity score (ISS) greater than 12 (i.e. major trauma), while earlier publications discuss the frequency of neurologic trauma among horseback riders with much less severe patterns of injury [3,11-21]. We have previously reported the basic epidemiology of major traumatic equestrian injuries [22], and now aim to expand upon the functional outcomes of patients.

The integral role of rehabilitation therapy in the recovery of patients who have sustained a major horse-related injury has not been previously described. The goals of this project were to (1) to define the incidence and pattern of severe equestrian trauma, (2) identify the current level of in-patient rehabilitation services, (3) describe functional outcomes for patients, and (4) discuss methods for increasing rehabilitation therapy in this unique population.

## Methods and results

Patients admitted to our institution as a direct result of equestrian injury with an ISS greater than 12, between January 1, 1995 and July 1, 2005, were identified via the trauma registry. The Foothills Medical Centre is the adult tertiary care trauma referral center for all major injuries in southern Alberta. This is also the area of the province where equine activities are most common. Each patient completed a 46-question telephone survey outlining the utility and functional outcome of their formal in-hospital rehabilitation therapy. The survey was developed by a group of clinicians including an occupational therapist, a physical therapist, trauma surgeons, recreational riders, and professional horseman. Questions were established from previous published literature, as well as from the authors' experience within the equestrian community and the rehabilitation setting. All participants were contacted by phone by an occupational therapist with personal horseback riding experience. Surveys lasted an average of 30 minutes, but ranged from 10 minutes to 1 hour. Patient and injury characteristics were identified from the trauma registry. Statistical analysis was performed using Stata version 8.0 (Stata Corp, College Station, TX.). Data were reported as means when normally distributed. Means were compared using the student's t test. Differences in proportions among categorical data were assessed using Fischer's exact test. A p value less than 0.05 was considered to represent statistical significance for all comparisons.

One hundred and fifty-one of 7941 (2%) trauma patients were injured while horseback riding (mean ISS = 20; operative rate = 45%; mortality rate = 6%) during the 10-year study period. Seventy-eight of 141 (55%) alive patients completed the survey. No patient refused to participate in the telephone interview. One patient, who was amnesic to the event and unable to answer questions accurately, had their spouse assist with the survey. The remaining patients were excluded because of outdated contact information.

### Pattern of severe equestrian trauma

The mean respondent was 47 years of age (range 20 to 78) and male (60%). The most common injuries were located in the upper body (Table 1). Sites of injury were chest (54%), head (48%), abdomen (22%) and extremities (17%). The mechanisms of injury included: 47 (60%) patients thrown from, or fell off a horse, 12 (16%) were crushed by a falling horse, 6 (8%) were kicked, 3 (4%) were stepped on, and 10 (13%) were injured by a variety of other mechanisms. Rider characteristics outlined a very experienced study cohort with a mean of 27 years riding experienced when injured. They typically rode Western style, for recreational and working purposes, on horses they owned. Forty-seven percent of all riders had been previously injured before their index accident and the minority reported the use of helmets or safety equipment, other than cowboy riding boots. Animal characteristics displayed an experienced and well-trained horse cohort. The horses were ridden often and had a median age of 7 years. Environmental factors identified the average injured rider as having ridden outdoors, in an open field, on dirt or uncultivated land, on a summer afternoon with good weather.

**Table 1:** Injury distribution

Chest	81 (54%)
Head	72 (48%)

Abdomen	33 (22%)

Skull Fractures	27 (18%)

Extremity Fractures	25 (17%)

Spinal Fractures	25 (17%)

Pelvic Fractures	23 (15%)

Spinal Cord	10 (6%)

Neck	2 (1%)

### Rehabilitation therapy

Thirty-eight (49%) patients received rehabilitation therapy. This included 46% physical and 18% occupational therapy. Injuries predictive of receiving therapy included extremity (73%), pelvic (80%) and spinal fractures (70%), as well as spinal cord trauma (100%)(Table 2). Having had a previous injury while horseback riding (47%) was predictive of not receiving therapy. Variables such as patient age, gender, ISS and years of riding experience were not statistically associated with participation in rehabilitation therapy while in hospital (Table 2). The prevalence of physical and occupational therapy services increased by 17% and 14% respectively, over the study period. Ten percent of all patients obtained additional therapy post-discharge (71% massage, 71% chiropractor, 29% acupuncture, 29% recreation therapy). When treated with in-hospital therapy, 25% obtained adjunctive outpatient therapy. When no in-hospital therapy was received, only 12% of patients sought outpatient therapy.

**Table 2:** Patient characteristics and provision of therapy

**Patient Characteristics**	**Received Therapy**	**Did Not Receive Therapy**
Mean Age (years)	48	47

Gender (% male)	51	49

Gender (% female)	46	54

ISS (mean)	22	19

Mean Riding Experience (years)	25	29

Previous Horse-related Injury (%)	33	62*

Abdominal Injuries (%)	47	53

Chest Injuries(%)	50	50

Extremity fractures (%)	73	27*

Head Injuries (%)	47	53

Neck Injuries (%)	0	100*

Pelvic Fractures (%)	80	20*

Skull Fractures (%)	8	92*

Spinal Cord Injuries (%)	100	0*

Spinal Fractures (%)	70	30*

ISS = Injury Severity Score

* = p < 0.05.

### Functional outcomes

Thirty-seven (55%) respondents had chronic physical difficulties as a result of the accident (50% male; 59% female). Seventy-six percent (28/37) of residual functional deficits were orthopedic related. Thirty-five percent (13/37) involved upper extremities, including pain and/or weakness in patient's hands, wrists or shoulders. Chronic pain or headaches were identified in 23 (62%) patients. Forty-one percent of respondents explicitly described functional disabilities involving decreased balance or limited use of hands and arms, including an inability to lift. In patients with head injuries, cognitive impairments included decreased memory, as well as mood and personality changes. Although 55% of respondents reported chronic physical difficulties, the majority (87%) were still riding at the time of the survey. Forty-six percent (31/67) did admit to changing their riding practices as a result of the injury however.

### Patient beliefs: Equestrian injury prevention & recovery

While some horseback riding injuries are a result of unpredictable events, 64% of riders believed the accident, and therefore their injury, was preventable. Riders with more experience faulted themselves, rather than the horse, for causing the injury. For example, several riders failed to adequately warm-up their horse or chose a riding setting with inherent risk such as a steep mountain trail. Participants admitted to asking their horse to perform a maneuver outside of its skill set in 21 (27%) cases. Respondents believed their horse had a bad temperament in 12 (15%) cases, was "spooked" in 27 (35%) instances, or simply lost their footing and fell in 9 (12%) cases. Environmental factors contributing to the accident were rarely identified. These included improper application, or failure of equipment such as saddles, cinches or halters in 6% of all injuries. Animals within the environment, such as another horse or dogs, were also infrequently reported as the cause of injury. Clearly, the majority of riders in this study believed that better decision-making might have prevented their injuries.

In general, participants had a strong desire to prevent similar accidents from happening to other riders, and were therefore willing to elaborate on many of the aspects of their accident and recovery. Several patients were thankful for the trauma department's interest in their recovery and described their participation in this study as cathartic. Desire for a support group following severe equestrian trauma was identified, along with offers to mentor fellow injured riders in their healing process.

## Discussion

Along with outdoor soccer and skiing, horseback riding is one of the three major sporting activities within the northern hemisphere most likely to result in long-term disability [10]. The risk factors identified for poor long-term outcomes include being an advanced rider and sustaining injuries other than fractures to the extremities [9]. It is therefore not surprising that 55% of our respondents reported long-term physical difficulties, considering the study cohort was an adult population with a mean of 27 years of riding experience. Furthermore, only 17% of these patients had limb fractures.

The rehabilitation therapy team assists patients in regaining skills and abilities lost as a result of injury, as well as aims to prevent, or minimize, long-term disability. Rehabilitation is defined as the act of restoring a patient to a condition of good health, such as the ability to work. For the severely injured equestrian population, this involves helping riders regain their physical independence and safety in everyday activities. Within the in-patient setting, this may include teaching patient transfers (bed, chair, bathtub, toilet, and car) and providing mobility training (walking, stair climbing). Activities of daily living such as bathing, dressing and eating can be addressed through strengthening, assistive device provision and instruction in adapted techniques.

In addition to physical rehabilitation, participants in our study also identified a need for psychosocial support following a severe equestrian injury. In-patient psychosocial care can be provided by therapists through encouraging participation in meaningful occupations during recovery and by assisting patients in identifying community supports in preparation for discharge from the hospital. Improving patient safety and independence following major trauma involves a team of physicians and therapists to adequately address a variety of patient needs. This team should include physiatrists, occupational therapists, physical therapists, speech and language pathologists and recreation therapists.

Rehabilitation therapy was significantly underutilized following severe equestrian trauma. The prevalence of physiotherapy and occupational therapy increased by 17% and 14% respectively, over the 10-year study period. Although reasons for the increase in patient access to rehabilitation services are speculative, they likely include an increase in therapist staffing on the trauma unit, as well as in the number of physician referrals to therapy services. With the majority of respondents experiencing chronic physical difficulties as a result of their accident, more rehabilitation therapy would likely improve long-term functional outcomes for the Western riding population.

This treatment resistant patient group was less likely to participate in therapy if they had sustained a previous horse-related injury. It is therefore important for therapists to acknowledge the priority placed on self-reliance within the Western riding culture. Once introduced to the scope and benefits of rehabilitation therapy however, equestrian injured patients willingly sought further assistance in their recovery effort, as 25% of respondents obtained adjunctive outpatient therapy.

Several strategies are available to increase the provision of rehabilitation services for this patient population. Referrals should be increased for underrepresented patient injuries that result in long-term disabilities. This includes brain, neck and skull trauma. Therapists must also discuss the functional deficits typically suffered by equestrian patients after discharge (balance deficits, upper extremity weakness, chronic pain, headaches, limited use of hands and arms, psychosocial challenges) with treating physicians. By explaining the potential benefits of therapy, even self-reliant Western riders are often willing to participate in rehabilitation activities.

This study has several limitations. Because patient participation in rehabilitation was reported and not observed, some responses may be biased. Second, our study had a moderate response rate of 55%. Although this is comparable with other telephone surveys using similar timeframes, the possibility of excluding a unique subgroup remains. Third, our institution is an adult trauma center that does not treat the 25% of injured Canadian riders younger than 16 years of age [1,3]. As a result, is important that these findings, specific to the severely injured Western horseback-riding patients, be confirmed in other rehabilitation centers.

## Conclusion

This is the first study to outline the provision, utility and functional outcomes of in-patient rehabilitation services in patients with severe equestrian trauma (ISS ≥ 12). Musculoskeletal and spinal cord injuries currently represent a large proportion of the targeted occupational and physical therapy services. Significant functional deficits are common after discharge from the hospital and therefore it is imperative that rehabilitation therapy be available to treatment resistant Western equestrian patients to reduce long-term impairment. Increased enrolment should target patients with brain, neck and skull injuries.

## Competing interests

The authors declare that they have no competing interests.

## Authors' contributions

All authors (JEB, CGB, RHM, ID, AWK) contributed to the conception of the study, analysis of data and completion of the manuscript. JEB was primarily responsible for obtaining telephone survey results/completion.
